# Laparoscopic Pancreaticoduodenectomy After Roux-en-Y Gastric Bypass: Case Report and Literature Review

**DOI:** 10.1155/cris/9982214

**Published:** 2025-05-20

**Authors:** Makai-Popa Silviu-Tiberiu, De Blasi Vito, Moga Marius Alexandru, Arru Luca, Goergen Martine, Azagra Juan Santiago

**Affiliations:** ^1^Department of General and Minimally Invasive Surgery, Luxembourg Hospital Center, Luxembourg, Luxembourg; ^2^Department of Surgery, “Regina Maria” Private Health Network, Brasov Hospital, Brasov, Romania; ^3^Faculty of Medicine, “Transilvania” University, Brasov, Romania

**Keywords:** bariatric surgery, pancreatic cancer, pancreaticoduodenectomy, Roux-en-Y gastric bypass

## Abstract

This is a case report of an alternate laparoscopic reconstruction possibility in a patient that required a cephalic duodenopancreatectomy (DPC) who previously underwent a Roux-en-Y gastric bypass (RYGB). The question of what type of reconstruction is to be performed in such patients is thoroughly debated in this article.

## 1. Introduction

Obesity is a “pandemic” of the twenty-first century reaching rates close to 18% in men and 20% in women in 2013 [1]. Its frequency is progressively increasing [2], and the surgical approach to treating obesity is also gaining ground, so it is logical to assume that the number of patients programmed for any other abdominal surgery with a Roux-en-Y gastric bypass (RYGB) in their surgical history might increase too. On the other hand, obesity is also a well-documented risk factor for cancer in general and for adenocarcinoma of the pancreas or cholangiocarcinomas in particular [3].

Logically, this all means that in the coming years, there will be more patients programmed for pancreatic resection after a RYGB which is why every useful information on such patients is, in our opinion, useful. This is the reason why we would like to present our reconstruction in a case of pancreatic head cancer, where the patient had previously benefited from a RYGB for morbid obesity.

## 2. Case Presentation

**Figure 1:**
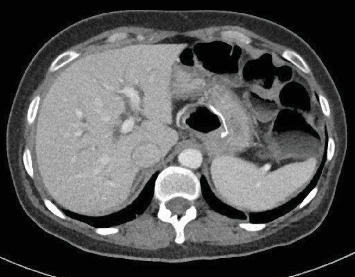
Abdominal computer tomography (CT) showing dilated extrahepatic and intrahepatic bile ducts.

**Figure 2:**
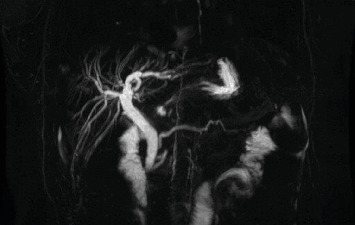
Magnetic resonance imaging (MRI) suspecting a potentially resectable distal cholangiocarcinoma.

A 39-year-old diabetic female patient, with a body mass index (BMI) of 39 kg/m^2^, underwent laparoscopic retrogastric, retrocolic RYGB in 2008. Following the RYGB, the patient dropped down to a BMI of 19 kg/m^2^ and had an adequate resolution of comorbidities. Ten years later, she presented in our department with a picture of obstructive jaundice (total bilirubin 6.4 mg/dL with a direct bilirubin level of 5.1 mg/dL). An abdominal computer tomography (CT) showed dilated extrahepatic and intrahepatic bile ducts (Figure 1), and a pancreatic magnetic resonance imaging (MRI) suspected a potentially resectable distal cholangiocarcinoma (Figure 2). An endoscopy was performed, but no tumor was visible, and a biopsy under echoendoscopy came back irrelevant. A laparoscopic duodenopancreatectomy (L-DPC) is programmed with curative intent. The patient was normoglycemic and had no other comorbidities at the time of cancer diagnosis.

**Figure 3:**
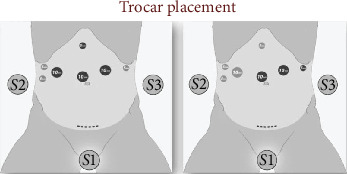
Trocar positioning.

**Figure 4:**
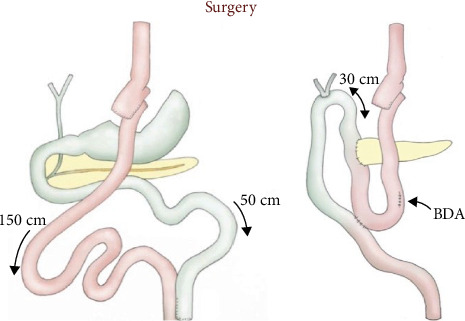
Schematic representation of the surgical reconstruction.

**Figure 5:**
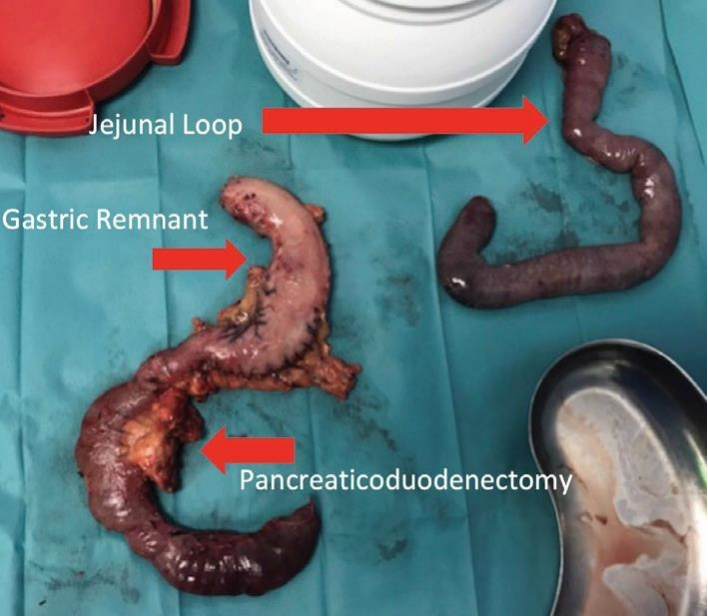
Resection specimen.

The patient was placed in a slight reverse Trendelenburg position, with the arms tucked alongside the body and legs spread. The main surgeon was placed between the legs, with one assistant on each side. By using four 5-mm trocars and three 10-mm trocars as shown in Figure 3,a full L-DPC was realized, with extraction of the specimen through a Pfannenstiel incision. Instead of making a classical reconstruction using three anastomosis and preserving the gastric remnant (GR), we decided to resect the biliary limb (BL) completely along with the specimen and the GR. For the reconstruction, we used the alimentary limb (AL), making both pancreatic and billiary anastomosis on the same limb in an Omega fashion, finishing with a mechanical side-to-side Braun jejunojejunostomy as shown in Figure 4. We performed the pancreaticojejunostomy (PJ) in a termino-lateral fashion using our classic technique of two continuous barbed suture layers [4]. The hepaticojejunostomy (HJ) was also performed in an end-to-side manner using two PDS 4/0 running sutures and tutorizing the anastomosis with an 8 Fr silastic tube. Regarding the Braun anastomosis, we performed it using a 60-mm motorized Echelon stapler with a white recharge. Blood loss was 300 cc, and the total operative time was 330 min. The postoperative period was uneventful, and the patient was discharged on day 10, resuming enteral nutrition on day 3 postoperatively. Amylase levels in the drains were monitored on days 1, 3, 5, and 9. The pathology of the resected specimen revealed an adenocarcinoma of the head of the pancreas (pT1aN0, 0/11 lymph nodes), instead of the initially suspected cholangiocarcinoma.  Figure 5 shows the resection specimens. Three months after surgery, a gastroscopy was performed due to an episode of melena, and we found we had an easy access to the biliary anastomosis due to our reconstruction.

No written consent for publication has been obtained from the patients as there is no patient identifiable data included in this case report.

## 3. Discussion

With the current rising number of bariatric surgeries and taking into account that obesity is a risk factor for pancreatitis and pancreatic cancer through different mechanisms, pancreatic surgeons will increasingly be asked to treat patients with a Roux-en-Y anatomy of their upper GI [3, 5–9].

The first challenge in periampullary or pancreatic lesions after malabsorptive bariatric surgery is diagnosis. Retrograde cannulation of the BP limb results in successful access to the ampulla in only 33% of patients with a low therapeutic success rate [10]. Deep enteroscopy techniques such as single-balloon and double-balloon enteroscopy or spiral-assisted enteroscopy and use of a long scope can achieve up to 80% access rate and 70% therapeutic success rate with a 3.4% complication rate [11, 12]. Surgically assisted techniques such as laparoscopic transgastric ERCP or endoscopy via a remnant gastrostomy tube have been described with success rates approaching 100% [11].

Nevertheless, according to an algorithm published by Peng et al. [13] for patients with resectable lesions, in cases that can be scheduled quickly for surgery—as in our case, biopsy is not necessary, and resection remains the most appropriate option.

The second challenge with such lesions after a RYGB lies in the specific technical aspects of pancreaticoduodenectomy (PD) after RYGB, and the debated aspects of management include minimally invasive versus open approach, resection of the defunctionalized GR, and type of reconstruction [13, 14]. A literature review describes, besides our case, two LPDs after RYGB, and one of these is a video presentation [14, 15]. In our opinion, progressive reduction of open duodenopancreatectomies could allow in more cases this kind of approach, especially for small tumors. In expert hands, LPD could be safe, improving hospital length of stay without augmenting operative time [4, 16].

Remnant gastrectomy is the favored management in literature series of duodenopancreatectomies in patients with a Roux-en-Y anatomy. Remnant gastrectomy eliminates one anastomosis and thus reduces postoperative morbidity related to ischemia, enteric leak, or delayed gastric emptying (DGE) and removes a potential site for future malignancy and bleeding [13–15, 17–21]. Only five cases out of a total of 28 in literature describes GR preservation (GRP), and two of the three major complications reported in the literature were in patients who did not undergo GR resection [13, 19, 21–24]. Even if GRP is considered due to advantages in terms of retained physiological function, nutritional support, and ease of future diagnostic and therapeutic interventions, literature analysis of cases shows that the decision of GRP is clearly due to secondary extensive adhesions and consequent difficult, safe dissection of the remnant [21, 23–25].

Nutritional advantages due to the risk of intolerance to oral intake could also be avoided by an intact GR, which provides the opportunity to leave a remnant gastrostomy tube for postoperative decompression and enteral nutrition post-PD [26]. There is also the question of the importance of GRP in terms of endocrine and exocrine functions, and the prevention of B12 deficiency could be a clear advantage of GRP [27].

Concerning GRP for future diagnostic and therapeutic interventions, it is clear that laparoscopic-assisted ERCP to access the remnant stomach, as opposed to the jejunum or gastric pouch, may provide a number of advantages, especially in terms of supporting access in a position similar to native anatomy [11, 12].

The type of reconstruction described in our case has the advantage of providing an easy biliary endoscopic access in case of relapses on the choledochal stump if a cholangiocarcinoma would have been the reason why the DPC was performed—as was our case initially. The easy access was confirmed during a gastroscopy for an episode of melena in the case of our patient.

Concerning the reconstruction, the use of prior BP limb for the HJ and PJ is the most commonly adopted due to decreased operative time and fewer anastomoses and because it avoids injury to the AL, but the disadvantage is that sometimes, the BL may not be long enough.

In our case, we used the long Roux-en-Y AL (Figure 4) passed in a retrocolic fashion. End-to-side anastomoses were created between this loop and the proximal pancreatic stump and hepatic duct. A side-to-side Braun enteroenteral anastomosis was associated in order to reduce the risk of enteric reflux contents into the biliary tree, which would increase the incidence of cholangitis.

Severely dense adhesions around the GR could make completion gastrectomy difficult, especially in patients who have had a complicated postoperative course after their initial bypass operation. In these patients, the reconstruction described by Theodoropoulos seems to be in our opinion a good option [24].

No differences about oncological outcomes seem to be present between these reconstruction techniques.

## 4. Conclusion

PD after RYGB is complex and rarely performed. For the reconstruction, a one-loop reconstruction can be done, without an increase in the number of complications for the patient as shown in this article.

## Data Availability

The data that support the findings of this study are available from the corresponding author upon reasonable request.
